# Successful application of dietary ketogenic metabolic therapy in patients with glioblastoma: a clinical study

**DOI:** 10.3389/fnut.2024.1489812

**Published:** 2025-02-18

**Authors:** Andreas Kiryttopoulos, Athanasios E. Evangeliou, Irene Katsanika, Ioannis Boukovinas, Nikolaos Foroglou, Basilios Zountsas, Angeliki Cheva, Vaios Nikolopoulos, Thomas Zaramboukas, Tomas Duraj, Thomas N. Seyfried, Martha Spilioti

**Affiliations:** ^1^Department of Neurology, Aristotle University of Thessaloniki, Thessaloniki, Greece; ^2^Division of Child Neurology, St Luke's Hospital, Thessaloniki, Greece; ^3^Department of Diet and Nutrition, Papageorgiou General Hospital, Thessaloniki, Greece; ^4^Bioclinic Thessaloniki Medical Oncology Unit, Thessaloniki, Greece; ^5^Department of Neurosurgery, Aristotle University of Thessaloniki, Thessaloniki, Greece; ^6^Department of Neurosurgery, St Luke's Hospital, Thessaloniki, Greece; ^7^Department of Pathology, Faculty of Medicine, Aristotle University of Thessaloniki, Thessaloniki, Greece; ^8^ISTODIEREVNITIKI S.A., Surgical Pathology and Cytopathology Laboratories, Thessaloniki, Greece; ^9^Department of Biology, Boston College, Chestnut Hill, MA, United States

**Keywords:** ketogenic, glioblastoma, diet, multiforme, metabolic, brain, tumor

## Abstract

**Introduction:**

Glioblastoma multiforme (GBM) ranks as one of the most aggressive primary malignant tumor affecting the brain. The persistent challenge of treatment failure and high relapse rates in GBM highlights the need for new treatment approaches. Recent research has pivoted toward exploring alternative therapeutic methods, such as the ketogenic diet, for GBM.

**Methods:**

A total of 18 patients with GBM, 8 women and 10 men, aged between 34 and 75 years participated in a prospective study, examining the impact of ketogenic diet on tumor progression. The pool of patients originated from our hospital during the period from January 2016 until July 2021 and were followed until January 2024. As an assessment criterion, we set an optimistic target for adherence to the ketogenic diet beyond 6 months. We considered the therapeutic combination successful if the survival reached at least 3 years.

**Results:**

Among the 18 patients participating in the study, 6 adhered to the ketogenic diet for more than 6 months. Of these patients, one patient passed away 43 months after diagnosis, achieving a survival of 3 years; another passed away at 36 months, narrowly missing the 3-year survival mark; and one is still alive at 33 months post-diagnosis but has yet to reach the 3-year milestone and is, therefore, not included in the final survival rate calculation. The remaining 3 are also still alive, completing 84,43 and 44 months of life, respectively. Consequently, the survival rate among these patients is 4 out of 6, or 66.7%. Of the 12 patients who did not adhere to the diet, only one reached 36 months of survival, while the rest have died in an average time of 15.7 ± 6.7 months, with a 3-year survival rate of 8.3%. Comparing the survival rates of the two groups, we see that the difference is 58.3% (66.7% versus 8.3%) and is statistically significant with *p* < 0.05 (0.0114) and X^2^ = 6.409.

**Discussion:**

The outcomes observed in these patients offer promising insights into the potential benefits of the ketogenic diet on the progression of glioblastoma multiforme when compared to those who did not follow the diet consistently.

## 1 Introduction

Glioblastoma (GBM) is the most common primary malignant tumor of the brain and central nervous system. It accounts for 14.5% of all central nervous system tumors and 48.6% of malignant central nervous system tumors (1). Despite surgical excision (total or subtotal), followed by adjuvant radiotherapy and chemotherapy with temozolomide, patients with newly diagnosed GBM have a median overall survival of 12–18 months, with <10% surviving beyond 5 years (2, 3). Additionally, GBM is an invasive tumor and usually recurs within 32–36 weeks of initial diagnosis, despite maximal treatment.

The almost universal relapse and poor long-term prognosis highlight the need for new treatment approaches. Thus, in recent years, the research community has turned to the discovery of alternative therapeutic strategies for GBM, such as dietary ketogenic metabolic therapy (KMT). Dietary KMT is defined as a synergistic precision nutrition approach, incorporating biomarker-driven ketogenic diets, fasting and fasting-mimicking diets, as well as other lifestyle interventions (4, 5). Monitoring of adherence should be performed by quantitative, unbiased biomarkers, such as the glucose-ketone index (GKI) (6). In particular, recent advancements in our understanding of cancer metabolism have led to renewed interest in Warburg’s theory of carcinogenesis (7–9). According to this theory, malignant cells are characterized by distinct structural and functional mitochondrial abnormalities, leading to compensatory metabolic dependencies. The main metabolic phenotype of GBM is a high glycolytic rate with lactic acid fermentation due to loss of efficiency in the respiratory cycle, despite ample mutational heterogeneity and secondary metabolic reprogramming (10). Unlike normal brain cells, that have evolved to metabolize ketone bodies for energy when glucose availability is low, GBM cells depend on glycolysis for growth and are unable to efficiently metabolize ketones due to impaired mitochondrial function (11–13). This metabolic deficiency isolates cancer cells from normal cells from a metabolic perspective, regardless of their somatic mutation landscape.

Based on the above observations, interest is growing in designing metabolism-based treatments for cancer in general and primary brain tumors in particular. Specifically, GBM cells lack metabolic versatility due to mitochondrial abnormalities and are largely dependent on glucose and glutamine for energy and biosynthesis (7, 8). Moreover, Warburg discovered that the excessive amount of glucose consumed by tumor tissues is fermented to lactate despite the presence of oxygen, rather than oxidized via mitochondrial respiration (6). However, it is still unknown to what degree other pure lyoxidative fuels, such as lactate, fatty acids, or ketone bodies, could contribute to cell survival and/or proliferation under relative glycolytic depletion *in vivo*. It is hypothesized that cancer cells cannot compensate for the simultaneous inhibition of glycolysis and glutaminolysis via ketone body metabolism due to acquired metabolic inflexibility (14). This hypothesis is strengthened by the presence of triglyceride-rich cytoplasmic lipid droplets in many malignant cancers. Recent research work has provided strong evidence that the presence of cytoplasmic lipid droplets and the aerobic fermentation commonly seen in most malignant cancers can serve together as biomarkers for oxidative phosphorylation inefficiency (15).

**Figure 1:**
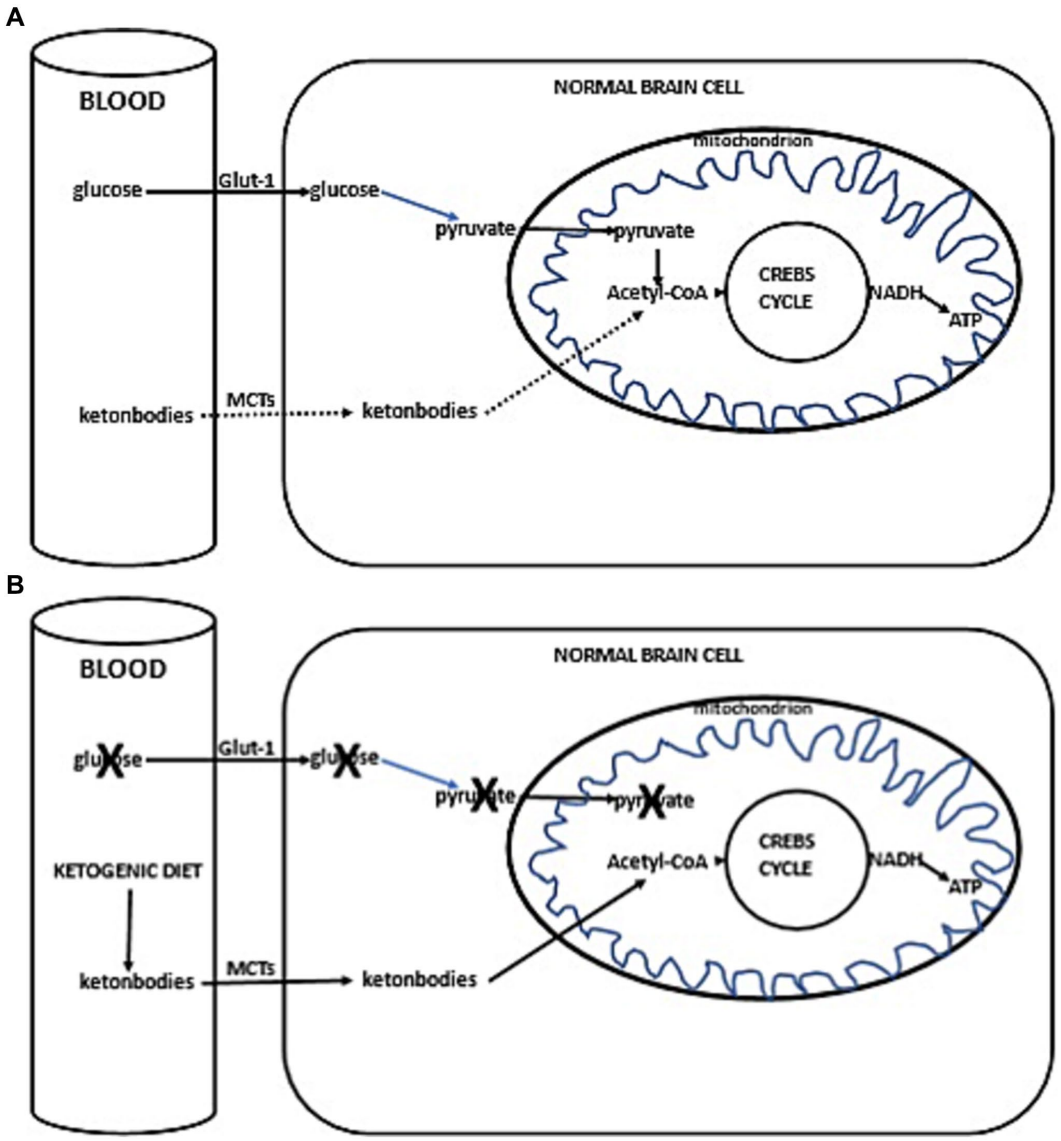
**(A)** Simplified scheme of glucose and ketone metabolism in a normal brain cell. Under anaerobic conditions, normal cells perform glycolysis in the cytoplasm, generating minimal but rapid energy. In aerobic conditions, normal cells perform the slower but more efficient oxidative phosphorylation in mitochondria for energy production. In a fed state, cellular energy is derived from glucose metabolism (illustrated with a solid line), undergoing glycolysis in the cytoplasm to form pyruvate, which then enters the mitochondrion. Inside the mitochondria, pyruvate is converted into acetyl-CoA, initiating the citric acid cycle (Krebs cycle), leading to the production of reducing equivalents. NADH/FADH are subsequently oxidized to generate ATP (solid line). In a fasted state, when glucose availability is low, the cell uses alternative ketone bodies that pass into the cell through monocarboxylate transporters (MCTs, indicated with a dotted line). Ketone bodies are converted into acetyl-CoA, prompting the citric acid cycle to proceed, similarly producing NADH (+H), which is oxidized to produce ATP (dotted line). **(B)** Ketogenic diet impact in normal brain cells. Due to the ketogenic diet-induced competition for available glucose, the primary source of acetyl-CoA switches to ketone bodies. These ketone bodies enter the cell via MCTs, leading to the production of acetyl-CoA. This initiates the citric acid cycle (Krebs cycle), resulting in the generation of NADH (+H). NADH is then oxidized, producing ATP.

**Figure 2:**
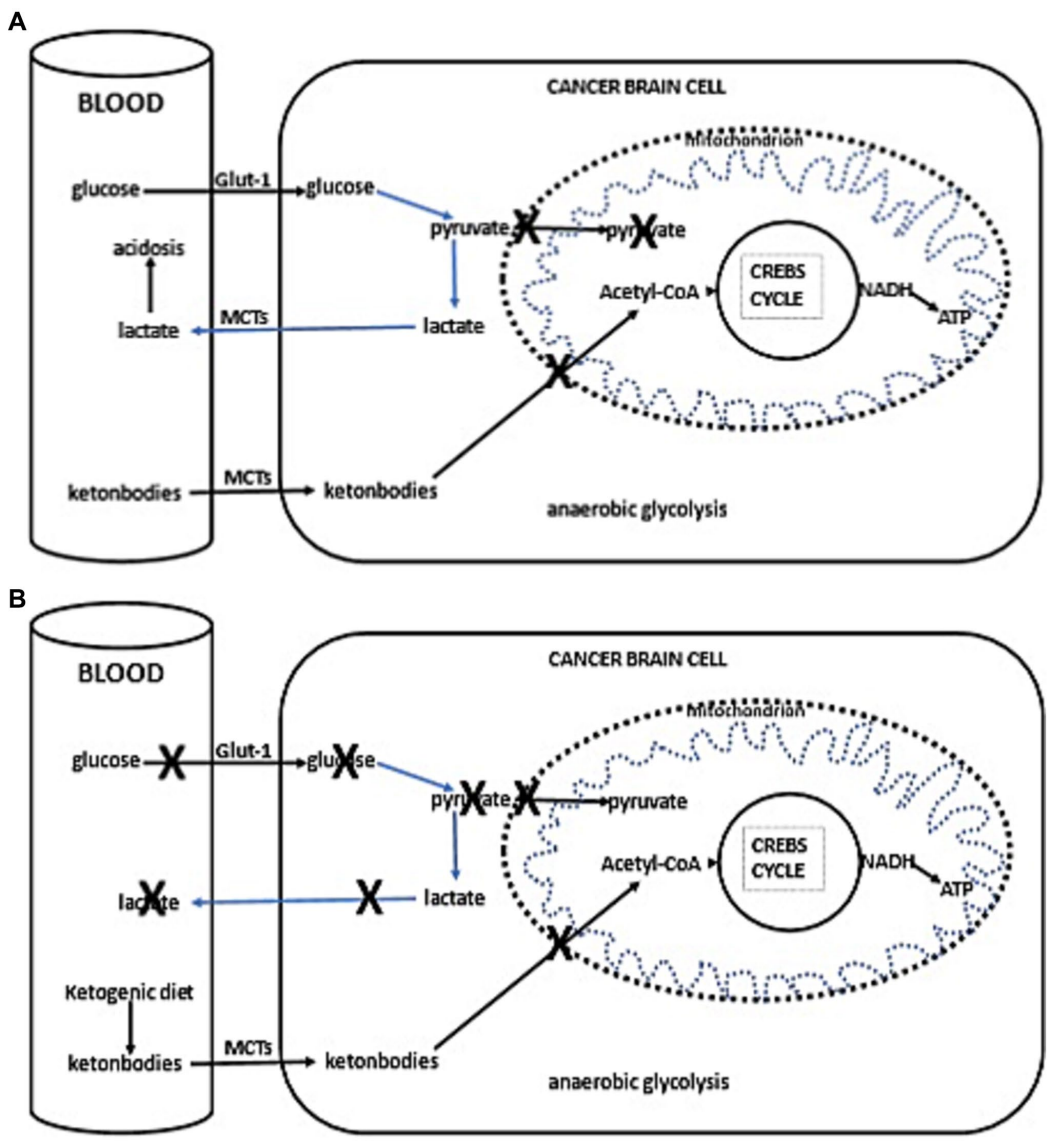
**(A)** Simplified schema of glucose and fat metabolism in a cancer cells. In addition to their reliance on glycolysis, most tumors, including those in the brain, exhibit abnormalities in the number and function of their mitochondria. Functional mitochondria are essential for utilizing ketones as an energy source. Consequently, for malignant cells, glycolysis becomes the primary source of ATP through the Embden–Meyerhof–Parnas pathway, regardless of oxygen availability. This glycolytic process is much less efficient than oxidative phosphorylation, as it generates less ATP per glucose molecule metabolized. Thus, conversion of glucose into lactic acid, bypassing oxidative phosphorylation, leads to reduced ATP production. To fulfill the elevated energy needs necessary for rapid tumor growth, cancer cells escalate glycolytic activity. The accumulation of lactate in cancer cells promotes lactate transport to the blood and extracellular fluid via proton-linked MCTs. This accumulation of lactic acid contributes to acidosis in both the blood and extracellular spaces, promoting angiogenesis, metastasis, and notably, immunosuppression, which is associated with worse clinical prognosis. **(B)** Ketogenic diet impact in cancer cells. The adoption of the ketogenic diet leads to an increase in hepatic ketogenesis, which in turn inhibits glucose uptake by cells, positioning ketones as the primary energy substrate. However, the reduced ability of cancer cells to oxidize ketones efficiently, compounded by glucose deprivation, may result in reduced proliferation rates.

Thus, with the current failure of chemoradiotherapy to improve long-term outcomes in GBM, as well as the proposed inability of GBM cells to efficiently oxidize ketone bodies when glycolysis is sufficiently limited, dietary KMT has been tested in the clinic to weaken the tumor and protect healthy cells during cytotoxic treatments (16, 17). Glucose and ketone metabolism in normal and cancer cells is illustrated in Figures 1A,B, 2A,B. While there is adequate experimental evidence supporting the beneficial effects of KMT on brain tumors, clinical testing is still ongoing (13, 18, 19). Most of the translational research to date involved case series and pilot clinical trials (20–24). In experimental models, KMT reduces glycolytic flux in cancer cells and increases ketolysis in non-tumoral cells, while also enhancing tumor-reactive immune responses (25). Therefore, biomarker-driven ketogenic diets could be a complementary therapeutic intervention for patients with GBM, aiming to slow tumor growth, potentiate standard cytotoxic therapies, and extend long-term survival.

Based on Warburg’s theory and expanding upon the abovementioned experimental and clinical data, we offered ketogenic diet therapy to a cohort of 18 GBM patients. GBM was chosen as a target due to well-characterized metabolic dependency upon glycolysis and poor prognosis despite maximal standard therapies (3, 26, 27). Our decision to offer ketogenic diet therapy to our patients was strengthened by the fact that no significant side effects have been reported in previous safety and feasibility studies (28, 29).

## 2 Materials and methods

### 2.1 Study design

A total of 18 patients with GBM, 8 women and 10 men, between 34 and 75 years of age (median age 57.5 years) participated in our prospective study evaluating the effects of ketogenic diet therapy on tumor progression. The pool of patients originated from our hospital and from a collaborating oncologist during the period from January 2016 until July 2021 and were followed until January 2024. The study was conducted in accordance with the1975 declaration of Helsinki and received prior approval from the ethics committee of our university (PN 1232/16). A double-blind design was planned, but due to the rapid progression of the disease, a survival of 3 years or more was considered as a success factor (30, 31). All subjects were referred to our hospital’s outpatient clinic for a general neurological assessment, including neurological status and MRI evaluation. A detailed laboratory evaluation was also performed, including the following: complete blood count, biochemical tests (electrolytes, blood glucose, transaminases, cholesterol, triglycerides, thyroid hormones), electrocardiogram, and electroencephalogram (EEG).

Each patient underwent a clinical assessment according to the ECOG (Eastern Cooperative Oncology Group) scale, before starting the ketogenic diet and every 3 months after. This scale is intended to assess how a patient’s disease is progressing, how the disease affects the daily living abilities, and to determine appropriate treatment and prognosis. Grade 0 means that the patient is fully active, able to carry pre-disease performance without restriction, while Grade 4 means that the patient is completely disabled; cannot perform any self-care; confined to bed or chair; in Grade 5, the patient is dead. Grades 2 and 3 are intermediary forms (32) (Table 1).

**Table 1:** Characteristics of all patients who participated in the study.

P	G	A	DD	DS-TOO	Chemo + Rad prior KD administration	MB
**1**	**M**	**41**	**29/12/16**	**4/1/17** **Total resection**	**30 cycles of radiation (02/17)** **Temozolamide**	**IDH 1 (−)**
**2**	**M**	**56**	**28/05/20**	**5/6/20** **Total resection**	**30 cycles of radiation (08/20)** **Temozolamide**	**IDH 1 (−)**
**3**	**F**	**64**	**19/04/21**	**26/4/21** **Total resection**	**30 cycles of radiation (05/21)** **Temozolamide**	**IDH 1 (−)**
**4**	**M**	**61**	**22/02/18**	**13/3/18** **Stereotactic biopsy**	**30 cycles of radiation (04/18)** **Temozolamide**	**IDH 1 (−)**
**5**	**M**	**48**	**24/04/20**	**4/5/20** **Total resection**	**30 cycles of radiation (06/20) Temozolamide**	**IDH 1 (−)**
**6**	**M**	**58**	**08/05/20**	**20/5/20** **Total resection**	**30 cycles of radiation (07/20)** **Temozolamide**	**IDH 1–2 (−)**
7	M	60	11/12/18	19/12/18 Subtotal resection	30 cycles of radiation (02/19) Temozolamide	IDH 1–2 (−)
8	M	69	15/03/17	23/03/17 Total resection	30 cycles of radiation (05/17) Temozolamide	IDH 1 (−)
9	M	53	1/10/20	15/10/20 Subtotal resection	30 cycles of radiation (11/20) Temozolamide	IDH 1 (−)
10	F	57	16/02/21	26/2/21 Total resection	30 cycles of radiation (04/21) Temozolamide	IDH 1 (−)
11	F	36	20/01/18	25/11/18 Subtotal resection	30 cycles of radiation (02/19) Temozolamide	IDH 1 (+)
12	F	44	08/09/17	19/9/17 Total resection	30 cycles of radiation (11/17) Temozolamide	IDH 1 (+)
13	F	34	24/07/20	3/8/20 Total resection	30 cycles of radiation (10/20) Temozolamide	IDH 1–2 (−)
14	F	59	08/01/16	22/1/16 Subtotal resection	30 cycles of radiation (03/16) Temozolamide	IDH 1–2 (−)
15	M	51	02/05/19	16/5/19 Total resection	30 cycles of radiation (06/19) Temozolamide	IDH 1–2 (−)
16	F	75	15/07/21	29/7/21 Total resection	30 cycles of radiation (09/21) Temozolamide	IDH 1 (−)
17	F	59	04/04/19	8/5/19 Subtotal resection	30 cycles of radiation (06/19) Temozolamide	IDH 1–2 (−)
18	M	71	22/01/20	24/2/20 Ttotal resection	30 cycles of radiation (04/20) Temozolamide	IDH 1 (−)

Patients 1–6 (bold letters) are those who maintained the diet beyond 6 months. P, Patient; G, Gender; A, Age; DD, Diagnosis date; DS-TOO, Date of surgery-type of operation; Chemo + Rad, Chemotherapy + Radiation; KD, ketogenic diet; MB, Molecular biology.

### 2.2 Inclusion–exclusion criteria

Adult patients, 18–75 years old with newly diagnosed glioblastoma multiforme (GBM) were included. Patients with cachexia (weight body<40 kg), with severe cardiovascular or renal disease, and inherited metabolic disorders for which ketogenic diet is contraindicated, were excluded from the study.

### 2.3 Ketogenic diet administration

Following this enrollment evaluation, consultation was done by a clinical dietician and then, if the patient agreed to participate, the ketogenic diet was initiated. Prior to initiating the diet, comprehensive hematological and biochemical testing was conducted. This included, among other evaluations, a full blood count, a complete lipid profile, and purine levels to prevent the risk of gout. We started with a 1:1 diet (fat: protein+ carbohydrates), and gradually, while monitoring ketonemia and glycemia, increased the ratio with the goal of reaching a 3:1 ratio, aiming for ketone values >3.5 mM/L and glucose values <80 mg/dL. Energy requirements were calculated based on the patients’ body weight at the time we took them on, using the Mifflin-St Jeor equation (which uses the current body weight). Our goal was not weight loss during the phase of standard therapy in these oncology patients However, we did observe that patients on the ketogenic diet initially lost weight and then stabilized. Thus, energy needs were calculated to maintain weight, though as previously mentioned, patients initially lost weight (on average 3–5 kg), regardless of minor increases in caloric intake. The highest ketogenic ratio we reached was around 2.5:1 since, in adults, achieving a higher ketogenic ratio while meeting daily protein requirements is challenging; these requirements were calculated to provide 0.95–1.2 g/kg/day. We did not apply caloric restriction for our patients,. Besides, we did not have obese patients, only a few who were overweight. The diet was adapted to Mediterranean diet patterns due to the patient’s aggravated status from the disease itself and standard treatments, with the intention of avoiding additional side effects. The Mediterranean ketogenic diet, unlike the western ketogenic diet that uses saturated fatty acids as a source of fat, is based mainly on olive oil and other mono and polyunsaturated fatty acid sources (olives, avocado, nuts, *ω*-3 rich fish) and emphasizes the consumption of ω-rich fish and seafood as a source of protein. This dietary pattern is milder than the classic North American-Northwestern. An example of the diet applied is shown in Table 2.

**Table 2:** Example of ketogenic diet 2,150 Kcal 2:1 ratio.

Morning	Snack	Lunch	Snack	Dinner
17 g tuna in oil	20 g feta cheese	93 g sardines	1 egg fortified with ω-3 fatty acids	117 g raw green salad
60 g keto-focaccia	8 g olive oil	41 g olive oil	15 g avocado	93 g salmon
15 g tahini	21 g olives	117 g boiled greens	9 g olive oil	41 g olive oil
14 g olive oil	25 g melon			
30 g avocado				

Daily menu plan.

### 2.4 Assessment criterion: statistics

As an assessment criterion, we set an optimistic target for adherence to the ketogenic diet beyond 6 months. The six-month timeframe was arbitrarily chosen based on our previous experience with studies involving the implementation of the ketogenic diet, where it served as an indicator of adherence (33, 34). We considered the therapeutic combination successful if the survival from diagnosis reached at least 3 years. The patients self-measured their blood glucose and ketone levels with blood glucose and *β*-ketone test strips, in the morning and afternoon preprandially. Initially, they did this daily for the first month and then twice a week. The readings were kept and referred to the dietitian with the diet records.

Statistical analysis was done using the Statistical Analysis Systems statistical software package, version 20 (SAS Institute). Results were regarded as significant when *p* < 0.05. The difference between the two groups was assessed by the *t*-test for paired comparisons.

## 3 Cases presentation

### 3.1 Patient 1: 84-months follow-up

**Figure 3:**
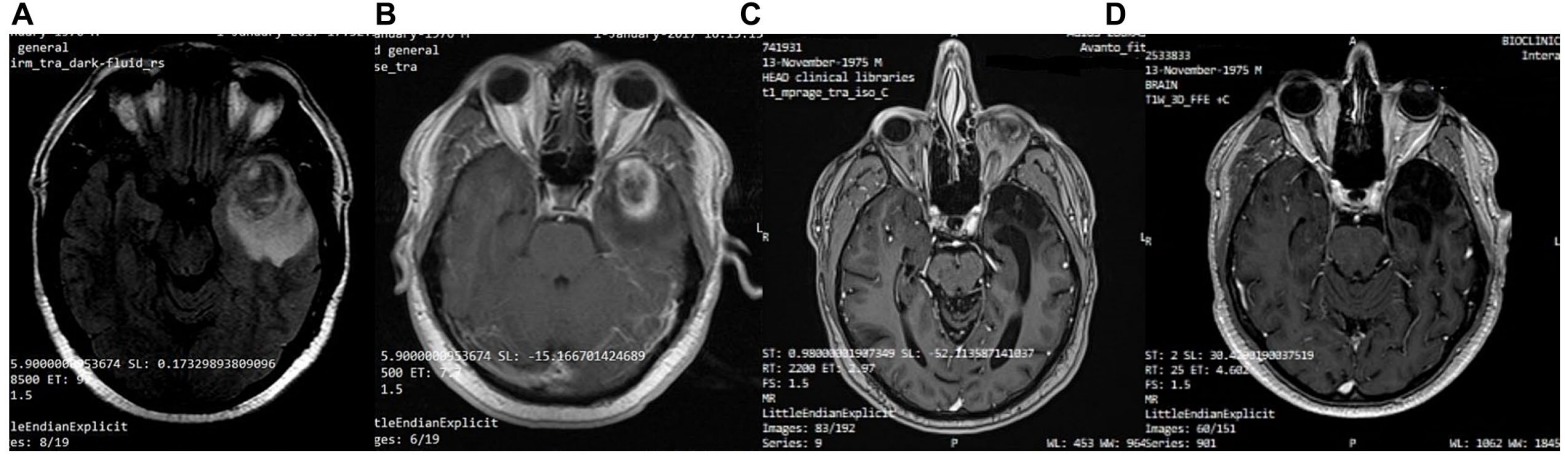
Patient 1: **(A)** Pre-operative brain MRI (T2/FLAIR) **(B)** Pre-operative brain MRI (T1 with contrast) **(C)** 38-month follow-up brain MRI (T1 with contrast) **(D)** 80-month follow-up brain MRI (T1 with contrast).

**Figure 4:**
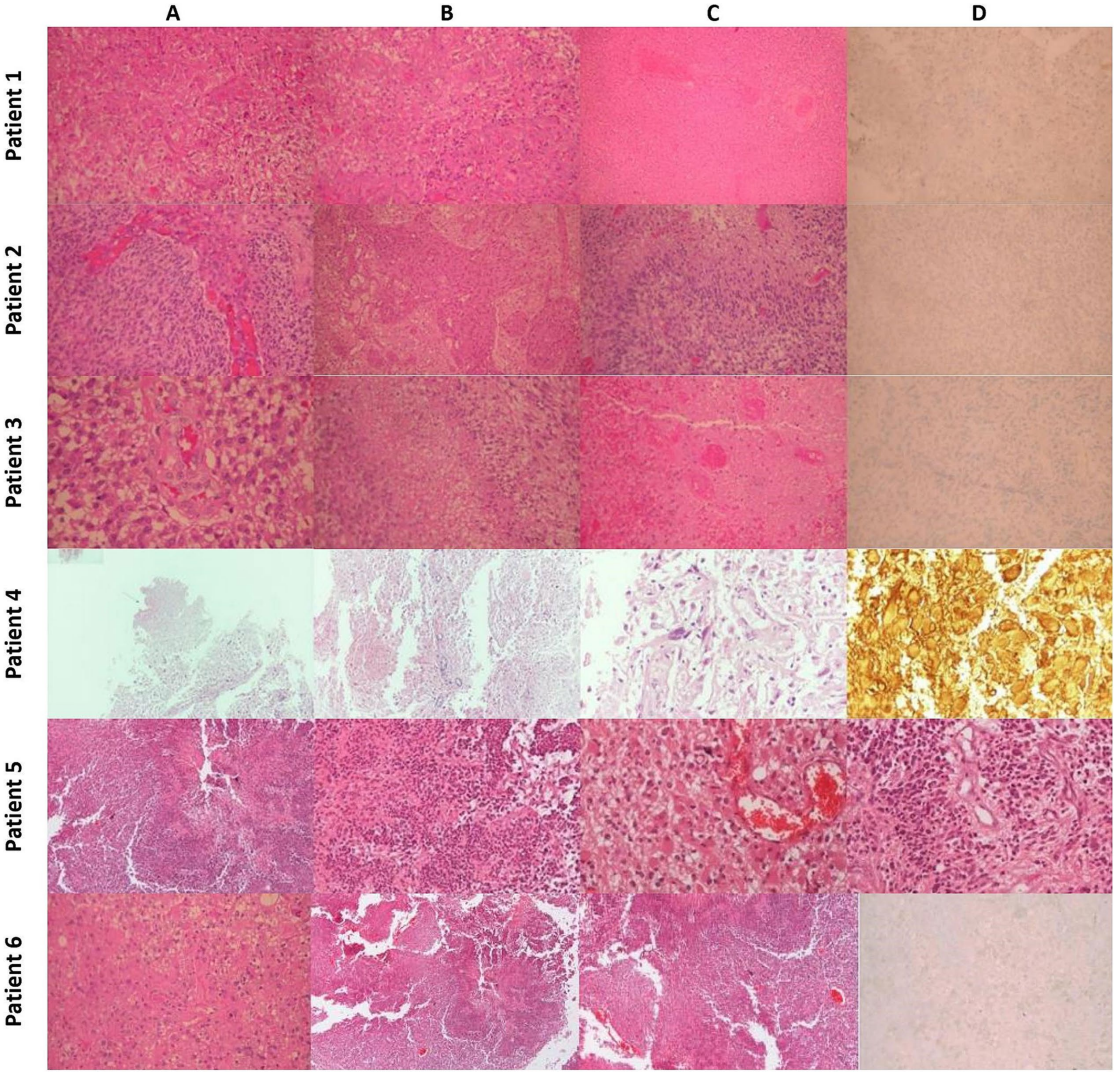
Histopathology: *Patient 1* **(A)** Typical Morphology of Glioblastoma (H&E x200)**. (B)** Significant endothelial hyperplasia (H&E x200). **(C)** Extensive Coagulative Necrosis with Thrombotic Vessels (H&E x100). **(D)** Tumor cells negative for IDH-1 Mutant (IHC x200). Patient 2: **(A)** Typical morphology of glioblastoma with endothelial hyperplasia (H&E x200). **(B)** Severe endothelial hyperplasia (X100). **(C)** Palisading necrosis (H&E x200). **(D)** Tumor cells are negative for IDH-1 mutant (IHC x200). *Patient 3* **(A)** Typical morphology of glioblastoma with endothelial hyperplasia (H&E x200). **(B)** Palisading necrosis (H&E x200). **(C)** Extensive coagulative necrosis with thrombotic vessels (H&E x200). **(D)** Tumor cells are negative for IDH-1 mutant (IHC x200). *Patient 4* **(A)** Region of tumor necrosis (H&Ex40). **(B)** Endothelial hyperplasia (H&Ex100). **(C)** Pleomorphic glial cells (H&E x400). **(D)** GFAP+ neoplastic cells and gemistocytes (H&Ex400). *Patient 5* **(A)** Region of tumor necrosis (H&E x40). **(B)** Neoplastic cells surrounding central necrosis (H&E x100). **(C)** Glial cells with gemistocytic features and microvascular proliferation (H&E x200). **(D)** Endothelial hyperplasia and microvascular proliferation (H&E x200). *Patient 6* **(A)** Typical morphology of glioblastoma (H&E x200). **(B)** Area of geographic necrosis (H&E x200). **(C)** Endothelial hyperplasia (H&E X200). **(D)** Tumor cells negative for IDH-1 Mutant (IHC x200).

A 41-year-old man was diagnosed with GBM of the left temporal lobe on December 2016 following a brain MRI (Figure 3). The presenting symptom was persistent headache and gradual word-finding difficulty (anomic aphasia). He underwent surgical resection in January 2017, followed by 30 sessions of radiotherapy (60 Gy) along with chemotherapy (temozolomide). Histopathological examination was typical of GBM with IDH1-*negative*/*MGMT*-*non-methylated* (Figure 4). On March 2017, the patient was started on a calorie restricted 1.4:1 ketogenic diet as adjunctive therapy (Table 3). Blood ketones and glucose levels were self-monitored daily. The patient achieved adequate ketosis during the first week of instituting the ketogenic diet and maintained high ketone levels (3–4 mmol/L) and adequate blood glucose levels (60–90 mg/dL) throughout the observational period. The ketogenic diet was well tolerated, with only mild gastrointestinal side effects (constipation). In addition, he received temozolomide, initially dosed at 100 mg/m^2^/day every other week. After 1 month, his dose increased to 200 mg/m^2^/day on the same schedule, without toxicities. Temozolomide maintenance treatment lasted for 2 years. Serial MRI brain imaging was obtained every 4 months. His follow-up brain MRI 79 months after diagnosis shows no evidence of tumor recurrence (Figure 3). The patient reports a residual mild anomia and is currently working as a teacher. His most recent ECOG grade is 0.

**Table 3:** Patient’s Ketogenic Diet: Total daily intake.

Patient-KD	Duration	Kcal	Fat g/d	Protein g/d	CHOs g/d	KR
Patient 1 Modified Ketogenic Diet	25.4.2017–present date	2000	169	100	20	1.4: 1
Patient 2 Mediterranean ketogenic diet with MCTs*	1st diet (19.8.2020)	2,150	189.2	73.9	19.6	>2: 1
	2nd diet (02.11.2021)	2,195	201.7	73.9	19.6	>2,2: 1
	3rd diet	2,298	213	75	20	>2,2: 1
	*The patient receives in total 150 g of MCT oil divided up into equal portions, as part of meals.					
Patient 3 Mediterranean Ketogenic Diet	05.7.2021 – present date	1,500	136 g/d	57 g/d	13 g/d	>2: 1
Patient 4. Mediterranean ketogenic diet with MCTs*.	1st diet (25.4.2018)	2,000	181.9	73	17.6	2:1
	2nd diet (20.09.2019)	2,125	195.5	73.2	18	>2:1
	*The patient received >5gr of MCT oil before bedtime.					
Patient 5 Mediterranean Ketogenic Diet	27.4.2020 – present date	2,000	189 g/d	65 g/d	11 g/d	2.5: 1
Patient 6 Mediterranean Ketogenic Diet	08.11.2020 05.05.2023	2,000	166 g/d	75 g/d	25 g/d	>2.5: 1

Fats are based mainly on olive oil, avocado, olives, raw nuts and seeds and proteins derive mainly from fatty fish and chicken. MCTs, Medium-chain triglycerides; CHOs, Carbohydrates; KD, Ketogenic diet; KR, Ketogenic ratio.

### 3.2 Patient 2: 43-months follow-up

**Figure 5:**
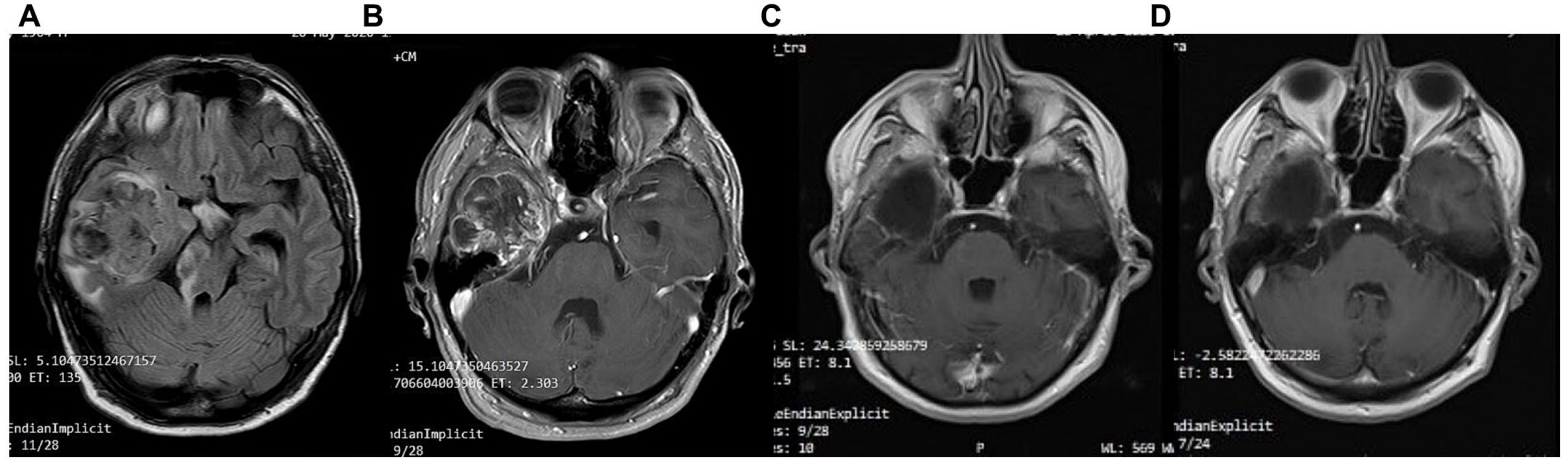
Patient 2: **(A)** pre-operative brain MRI (T2/FLAIR) **(B)** pre-operative brain MRI (T1 with contrast) **(C)** 20-month follow-up brain MRI (T1 with contrast) **(D)** 40-month follow-up brain MRI (T1 with contrast).

Patient 2 is a 59-year-old male. He is a primary school teacher in very good general condition, with a lean and athletic build (marathon runner). On 23/05/2020, at the age of 56, he was hospitalized due to persistent headache and a sudden onset of left hemiparesis, confusion, and vomiting. Brain CT showed a space-occupying right parietal–temporal lesion with solid and cystic elements and peripheral enhancement, as well as large perifocal edema with midline shift of 12 mm. A subsequent brain MRI (Figure 5) showed a large heterogeneous mass in the right temporoparietal area, 53 mm in diameter, with hemorrhagic and necrotic elements and peripheral gadolinium enhancement, surrounded by extensive vasogenic edema. These radiological features were highly suggestive for GBM. On 05/06/2020, he underwent a total resection of the tumor through a right temporal craniotomy. He was discharged with levetiracetam 1,000 mg × 2/day and methylprednisolone per os in gradual tapering. Histological examination (Figure 4) confirmed GBM, immunohistochemically negative for IDH-1 mutation (GBM NOS). On 28/06/2020 he suffered a lower extremity deep vein thrombosis (DVT) and was placed on a therapeutic dose of heparin for 8 months. The patient followed 30 cycles of radiation (21/7/2020–31/8/2020) and was placed on temozolomide 150 mg/m^2^, 5 days/month, which continues until the present day. A classic ketogenic diet was implemented on 19/8/2020 with a ketogenic ratio > 2:1 and a total daily calorie intake of 2,150 kcal (Table 3).

The patient achieved satisfactory ketosis as early as the 1st week of diet initiation, with blood ketone levels of 2.9–5 mmol/L and morning blood sugar of approximately 72–75 mg/dL. Nutritional ketosis is maintained until today. Close monitoring with serial MRIs shows no evidence of GBM recurrence throughout the observation period of 43 months (Figure 5). The patient is fully ambulatory, still working as a primary school teacher, with no imaging or clinical signs of disease activity. His most recent ECOG grade is 0.

### 3.3 Patient 3: 33 months follow-up

**Figure 6:**
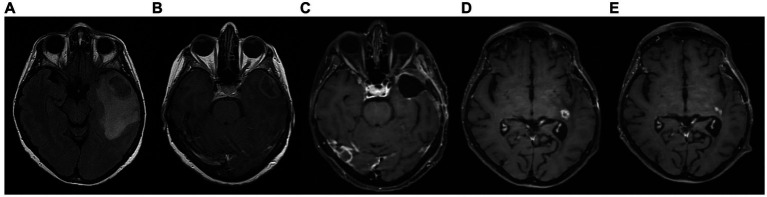
Patient 3: **(A)** brain MRI on diagnosis (T2/FLAIR) **(B)** brain MRI on diagnosis (T1 with contrast) **(C)** 9-month follow up brain MRI (T1 with contrast) **(D)** GBM relapse, 12-month follow up brain MRI (T1 with contrast) **(E)** 30-month follow up brain MRI (T1 with contrast).

A 65-year-old female was diagnosed with GBM in April 2021. Her presenting symptom was anomic aphasia. Brain MRI was remarkable for a space-occupying lesion at the left temporal lobe with heterogenous signal on T2 sequences and irregular enhancement surrounded by large vasogenic oedema, consistent with GBM (Figure 6). Her histopathological examination confirmed IDH1-negative GBM (Figure 4). She underwent surgery (total resection of the tumor) followed by radiotherapy—a total of 60 Gy in 30 RTs - and maintenance chemotherapy with temozolomide 120 mg/m^2^/day, for 5 days each month. Ketogenic diet with a ketogenic ratio of >2:1 and total daily calorie intake of 1,500 kcal/day was introduced in July of the same year (Table 3). After 6 months the patient decided to abandon the diet due to perceived dietary restrictiveness. On April 2022, 4 months after KD discontinuation, GBM recurrence was followed by stereotactic radiosurgery (CyberKnife) (Figure 6). She also initiated second line chemotherapy with bevacizumab (5–7 mg/kg) and irinotecan 120 mg/m^2^ twice/month, which is maintained until the present day. After relapse, the patient agreed to reinitiate the diet with improved adherence. Blood glucose levels were maintained between 75 and 85 mg/dL and ketone levels between 2 and 3 mmol/L. Her present ECOG grade is 0.

### 3.4 Patient 4: 43-month follow-up

**Figure 7:**
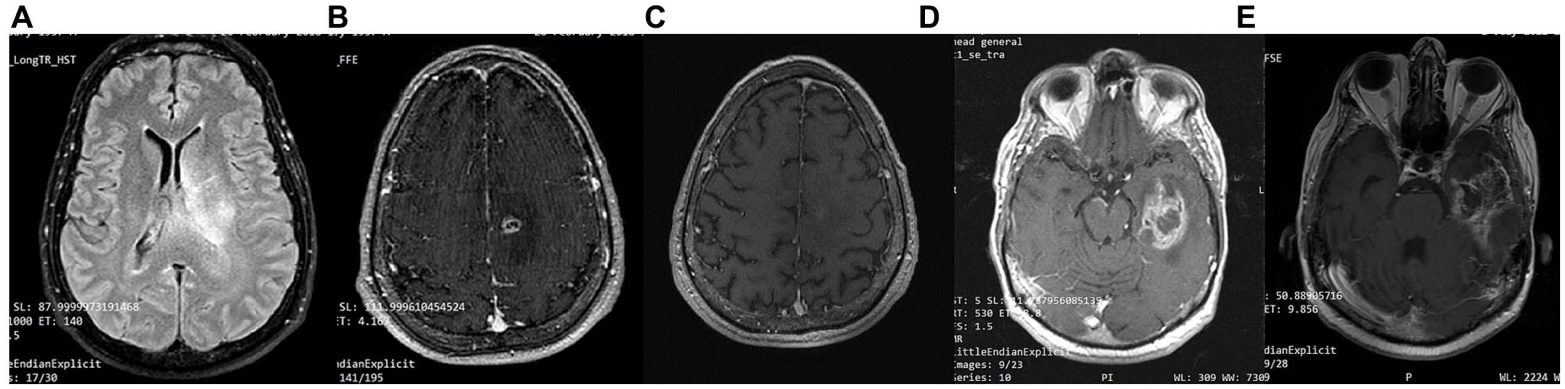
Patient 4: **(A)** brain MRI on diagnosis (T2/FLAIR) **(B)** brain MRI on diagnosis (T1 with contrast) **(C)** 24-month follow-up brain MRI (T1 with contrast) **(D)** 32-month follow up brain MRI. GBM relapse (T1 with contrast) **(E)** 41-month follow up brain MRI (T1 with contrast).

A 63-year-old man was diagnosed in February 2018 with GBM following a first epileptic seizure. The patient experienced an episode of focal seizures involving the right upper and lower limb. On neurological examination, a mild right-sided hemiparesis was noted. Brain MRI revealed a large heterogeneously enhancing tumor in the left parietal lobe with a central nodule and surrounding vasogenic oedema, extending through the corpus callosum to the contralateral side (Figure 7). An EEG demonstrated paroxysmal activity consisting of intermittent sharp theta waves on the left frontocentral region and the patient was put on levetiracetam. Stereotactic brain biopsy and histopathological examination confirmed IDH1-*negative* GBM (Figure 4). The patient was considered inoperable due to the extent of the tumor, which infiltrated the corpus callosum and extended across the midline. The patient received radiotherapy (30 sessions, 60 Gy in total) along with chemotherapy (temozolomide) and corticosteroids (dexamethasone per os). A 3:1 ketogenic diet was initiated in April 2018 just before radiotherapy (Table 3). After radiation therapy, corticosteroids were gradually withdrawn and the patient was maintained on a treatment regimen of 250 mg temozolomide in total per day for 5 days every 4 weeks (until July 2019), alongside ketogenic diet therapy. The diet was well-tolerated, and the patient achieved and maintained ketosis (ketonemia 3–4 mmol/L) and lower blood glucose levels (70–85 mg/dL) throughout the follow-up period. Brain MRI was performed every 3 months showing no tumor progression and less surrounding vasogenic oedema, while the enhancing nodule in the left semioval center was decreasing with minor enhancement (Figure 7).

However, 32 months after diagnosis, the patient suffered a GBM relapse (Figure 7). While continuing the ketogenic diet, he underwent surgical resection and was put on second line chemotherapy with bevacizumab (5–7 mg/kg) and irinotecan (120 mg/m^2^) until his death in September 2021 (43 months post-diagnosis).

### 3.5 Patient 5: 44 months follow-up

**Figure 8:**
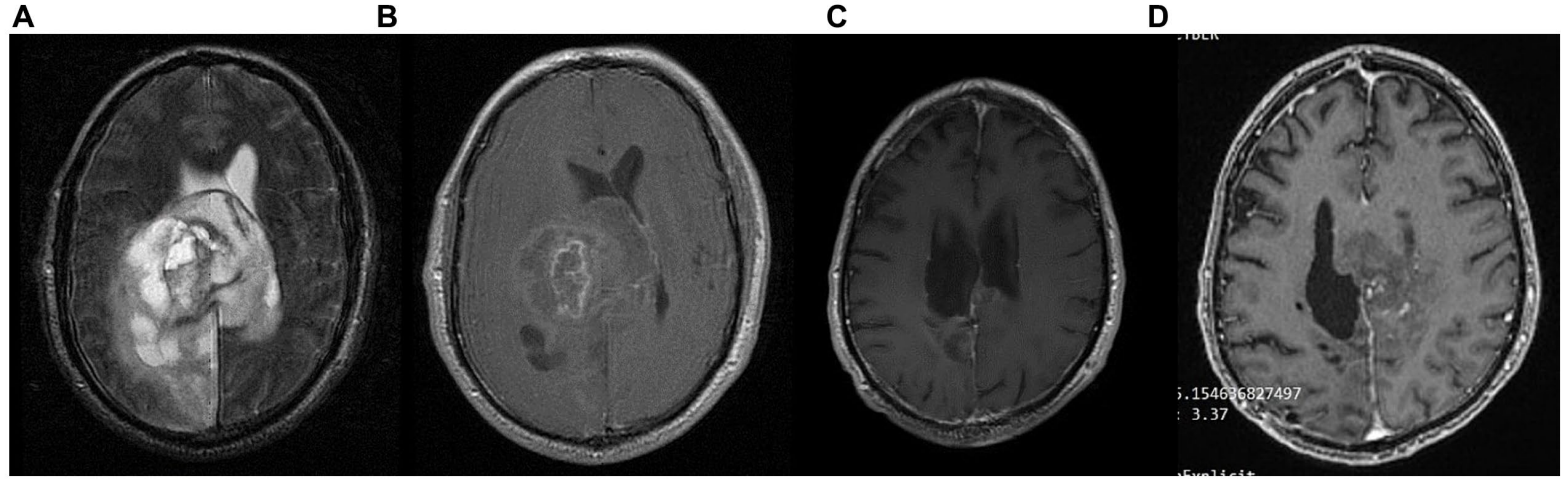
Patient 5: **(A)** pre-operative brain MRI (T2/FLAIR) **(B)** pre-operative brain MRI (T1 with contrast) **(C)** 34-month follow up brain MRI (T1 with contrast) **(D)** GBM relapse 40-month follow up brain MRI (T1 with contrast).

A 48-year-old male guitar teacher presented at the emergency department in April 2020 with confusion, agitation, anomic aphasia, and left hemiparesis. His symptoms developed gradually over a period of 1 month, during which he complained of frequent headaches. Brain MRI demonstrated a large contrast enhancing right hemispheric lesion expanding contralaterally through corpus callosum, as well as supratentorially to the right cerebellum, with irregular borders, necrotic and hemorrhagic areas and vast perilesional vasogenic oedema (Figure 8). These features were consistent with GBM, as was later confirmed by histopathological examination that showed grade IV IDH1-negative GBM (Figure 4). Ketogenic diet was introduced soon after diagnosis, as an adjunctive treatment to standard of care (tumor resection, radiotherapy, chemotherapy). Anticonvulsive treatment (levetiracetam) was also started due to focal aware motor seizures. The patient was put on classic ketogenic diet (ketogenic ratio of 2.5:1) with MCT supplementation, with a total caloric intake of 2000 kcal/day, containing 189 g/d fat, 65 g/d proteins, 11 g/d carbohydrates and 3–4 g MCT per meal (Table 3). The patient achieved and maintained ketosis (ketone levels 3–4 mmol/L) and lower blood glucose levels (70–85 mg/dL) soon after KD initiation. As a side effect during KD, the patient reported chronic constipation that was relieved by conservative or pharmacological means. During follow-up, blood ketone levels oscillated between 2–3 mmol/L and blood glucose ranged from 80 to 90 mg/dL.

The patient had no evidence of disease progression for almost 3 years, having a mild residual left hemiparesis and therefore being able to carry lighter everyday activities (ECOG grade 1). However, in May 2023 (36 months post diagnosis), the patient suffered a GBM recurrence (Figure 8) and was initiated with second line bevacizumab (5–7 mg/kg) and irinotecan (120 mg/m^2^) every other week. He is currently under CyberKnife treatment (4–5 sessions over 1–2 weeks). His present ECOG performance status is 3 (capable of only limited self-care; confined to bed or chair more than 50% of waking hours).

### 3.6 Patient 6: 36 months follow-up

**Figure 9:**
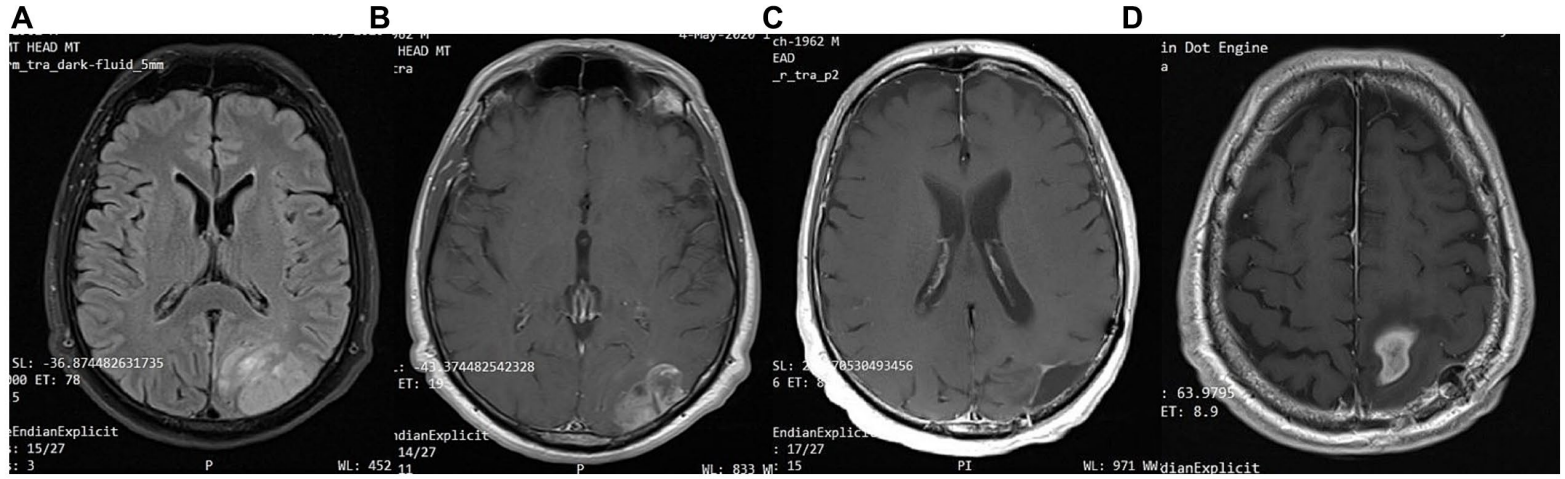
Patient 6: **(A)** brain MRI on diagnosis (T2/FLAIR) **(B)** brain MRI on diagnosis (T1 with contrast) **(C)** 18-month follow up brain MRI (T1 with contrast) **(D)** 24-month follow-up brain MRI (T1 with contrast).

A 58-year-old priest presented with neuro-psychiatric symptoms in April 2020, such as headaches, personality changes and aggressiveness. For this reason, he underwent a brain MRI, which revealed a space-occupying lesion on the left occipito-parietal region with heterogeneous enhancement (Figure 9). The patient underwent total excision of the tumor on May 20, 2020. Histological examination demonstrated GBM immunohistochemically negative for IDH-1&2 mutation (GBM NOS). The patient then underwent 30 sessions of radiation therapy (60 Gy) in combination with temozolomide (75 mg/m^2^) and dexamethasone. Subsequently, the patient received maintenance chemotherapy with temozolomide 150 mg/m^2^ for 21 months (Figure 9). In November 2020, 6 months after the diagnosis, he started a classical ketogenic diet with a ketogenic ratio > 2.5:1 (Table 3), which was well-tolerated, and the patient maintained low blood glucose levels (75–90 mg/dL) and satisfactory ketosis.

In February 2022, 22 months after the diagnosis, the patient experienced a focal epileptic seizure and was diagnosed with a recurrence of the GBM on a subsequent brain MRI (Figure 9). For this reason, he underwent 3 sessions of stereotactic radiotherapy (CyberKnife), and the patient was placed on second-line chemotherapy with bevacizumab (5–7 mg/kg) and irinotecan 120 mg/m^2^ twice a month. He continued the ketogenic diet but ultimately passed away from disease complications in May 2023, 36 months after the diagnosis.

## 4 Results

**Figure 10:**
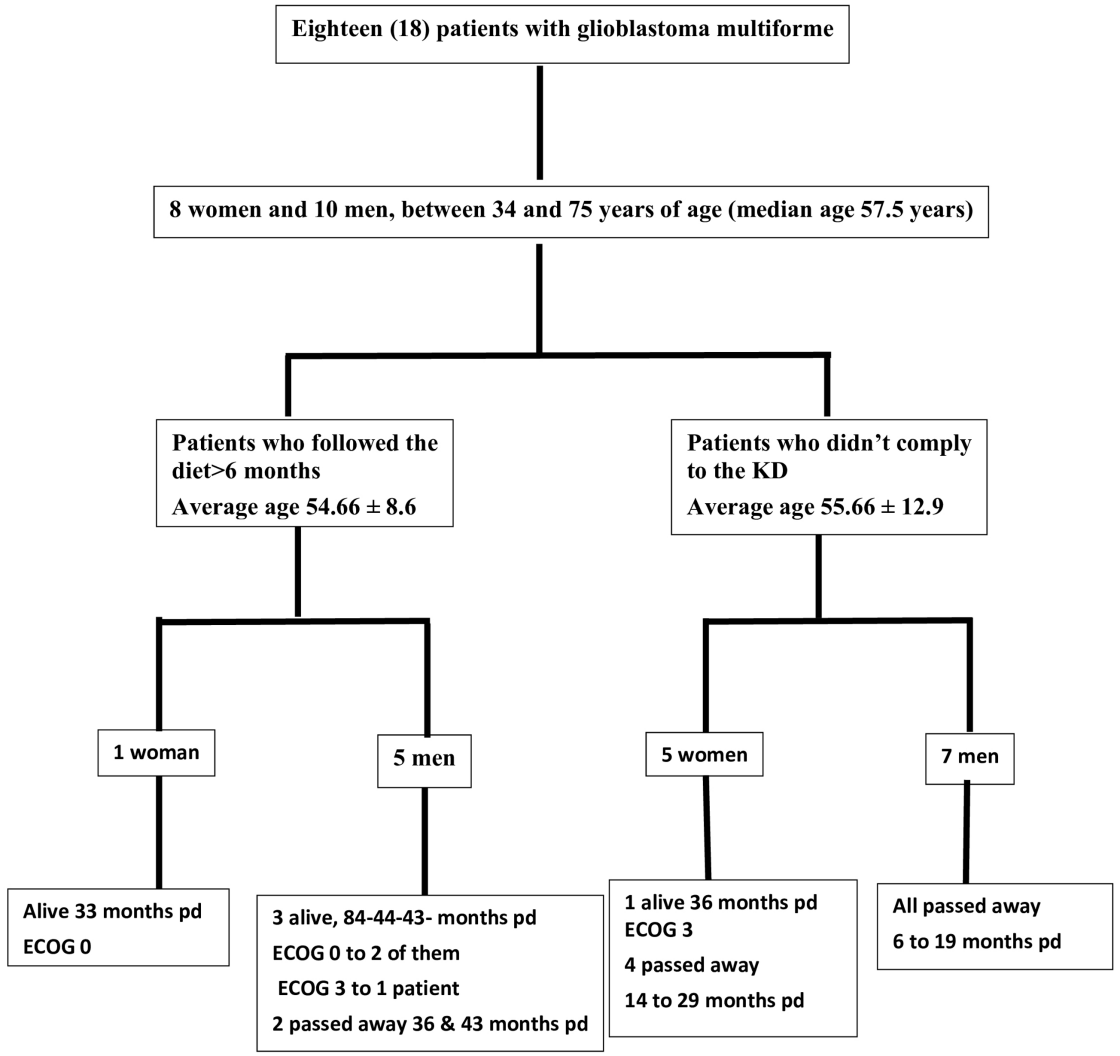
Study flow chart. pd, post diagnosis; ECOG, Eastern Cooperative Oncology Group.

Out of the 18 patients, 6 followed the diet for more than 6 months (Figure 10). The effect of diet on the disease evolution of these patients is shown in Table 4. More specifically, from the 6 patients who followed the diet for more than 6 months, one patient died at 43 months (thus achieving 3 years survival), one patient died exactly 36 months after diet initiation (thus narrowly missing 3 years survival), and one patient is still alive 33 months after the start of the diet (but has not yet reached the 3-year goal, and is not included in the final percentage). The remaining 3 are also still alive, completing 84, 43, and 44 months of life, respectively. Therefore, the 3-year survival rate is4/6 = 66.7%.

**Table 4:** Patients who followed the diet beyond 6 months.

Patient	DD	KD initiation	KD maintenance	Disease progression	GKI	Survival	Most recent ECOG Grade
P1	29/12/16	05/03/17	82 months	No evidence of disease progression	1.09–2.78	Still alive 84 months	0
P2	28/05/20	19/08/20	41 months	No evidence of disease progression	0.83–2.1	Still alive 43 months	0
P3	19/04/21	05/07/21 Stopped on January 22 Reinitiated May 22	In total 27 months	10/4/22 GBM relapse Cyberknife Reiniated KD	1.46–3.7	Still alive 33 months	0
P4	22/02/18	25/04/18	40 months	10/20 GBM recurrence -surgical resection – 15 cycles of radiation -Avastin	1.26–2.36	Died 8.9.2021 43 months	5
P5	24/04/20	27/04/20	40 months	Relapse	1.17–2.93	Still alive 44 months	3
P6	08/05/20	08/11/20	22 months in total	8/2/22 cyberknife Avastin	1.35–3.6	Died 36 months	5

P, Patients; P1-6, Patients whose numbers correspond to those in Table 1. DD, Diagnosis Date; GKI, glucose-ketone index; ECOG, Eastern Cooperative Oncology Group; ECOG Grade 0 = Patient fully active, able to carry on all pre-disease performance without restriction, 1 = Restricted in physically strenuous activity but ambulatory and able to carry out work of a light or sedentary nature, e.g., light house work, office work, 2 = Ambulatory and capable of all self-care but unable to carry out any work activities; up and about more than 50% of waking hours, 3 = Capable of only limited self-care; confined to bed or chair.

The characteristics of the patients who did not comply with the diet beyond 6 months are shown in Table 5. Of the 12 patients who did not adhere to the diet, only one reached 36 months of survival, while the rest have died in an average time of 15.7 ± 6.7 months, with a 3-year survival rate of 8.3%.

**Table 5:** Survival of patients who did not follow the diet.

Patient	Gender	Age	Overall survival
P7	M	60	12 months
P8	M	69	6 months
P9	M	53	14 months
P10	F	57	14 months
P11	F	36	25 months
P12	F	44	29 months
P13	F	34	Still alive 36 months
P14	F	59	17 months
P15	M	51	18 months
P16	F	75	12 months
P17	F	59	19 months
P18	M	71	11 months

Patients 7–18 are therefore the same patients as numbered in Table 1.

Comparing the survival rates of the two groups, we see that the difference is 58.3% (66.7 versus 8.3%) and is statistically significant with *p* < 0.05 (0.0114) and X^2^ = 6.409.

Unique features were observed in the 6 patients who adhered to the diet (Table 3). Patient 1 (P1) received a combination of radiation and 75 mg/m^2^ temozolomide for 1 month. He continued with maintenance temozolomide at 180 mg/m^2^, 5 days/month for 24 months and then discontinued it. In total, he received concurrent chemotherapy + KD for 24 months. Only KD was administered in the remaining observation time (60 months). His ECOG performance status was excellent with score 0 (fully active). Patient P2 is alive at 43 months, with 36 months under ketogenic diet therapy with ECOG 1.Patient P3 stopped the ketogenic diet and relapsed soon after. She was initially treated with chemotherapy and cortisone without an increase in her blood glucose levels, and after the end of chemotherapy, she resumed the ketogenic diet and since then recovered with ECOG 1. All 6 patients received chemotherapy and radiation, as well as corticosteroids, simultaneously with the ketogenic diet. It is remarkable that during this time, glucose values did not typically rise above 80 mg/dL. Comparatively, glucose values were particularly elevated at end-of-life care while on concomitant chemotherapy and corticosteroids in non-adhering patients.

## 5 Discussion

In our cohort, out of the 18 patients, 6 followed the diet for the predefined period of >6 months. The effects of the ketogenic diet observed in these six IDH-wild type GBM patients who adhered to the diet are promising, especially when compared to the disease progression in patients who were unable to maintain the diet, as well as to historical controls (1, 35–37). Two of them died after having lived 43 and 36 months, a period considerably longer than the average lifespan of patients with GBM (patient 4 and 6 respectively). The remaining 4 are still alive, with one completing 84 months of life (patient 1), while the other three remain alive at 43, 33 and 44 months (patients 2, 3, and 5 respectively). It should be noted that all adhering patients (with the exception of patient 1) continued intermittent chemotherapy. Therefore, the first question that arises is whether the ketogenic state contributed to the longer survival due to its effects on cancer metabolism, by potentiating chemotherapy, or both. We believe that the metabolic effects of the diet contributed to improved outcomes because the patients that did not receive it, or were not able to adhere to it, still receiving other standard of care treatments, exhibited shorter survival in our cohort.

When considering the patients as a whole, there was no significant difference in age between those who followed the diet and those who did not (average age of participants: 54.66 ± 8.6, non-participants: 55.66 ± 12.9).

There was concern regarding the significant number of patients who did not adhere to the diet, despite it being recommended to all and thoroughly explained, including its principles and expected outcomes. An attempt was made to uncover the reasons why these patients ultimately did not follow the diet. One potential reason for non-adherence to the diet in certain cases was the strong desire of close relatives to avoid discussing the seriousness of the diagnosis and the unfavorable prognosis with the patients. This applied to three of our patients who had already undergone surgical resection, radiation, and chemotherapy. They felt reassured that they had done everything possible and saw no need to adhere to a demanding diet. Another reason was a lack of trust in the diet; four of our patients did not believe that, following conventional therapies, a simple diet could be beneficial, despite initially agreeing to follow it. Older patients, in particular, struggled to adjust their eating habits and discontinued the diet shortly after starting. Additionally, two patients stopped the diet due to the absence of a supportive environment, while another two discontinued it due to financial constraints, as they found the ketogenic diet to be relatively costly to implement.

Another challenge encountered was the significant number of women who did not adhere to the diet. Out of the 8 women who participated, only one adhered to the diet, while the remaining 7 did not. While there are distinct differences in the pathophysiology of the disease between men and women, none of these differences can adequately explain these findings. Sex differences in glioblastoma patients have been well documented, with evidence suggesting that women tend to respond better than men to conventional treatments for this disease (38–40). Could these differences influence the effect of the ketogenic diet in the patients of this study? The number of participants is small, and among the 6 patients, only one is a woman. Larger studies, separating men and women, will certainly be necessary. Regarding the non-participants, it is interesting to note that women appear to have a longer life expectancy than men. However, analyzing this data is beyond the scope of the current study. Furthermore, the relatively small sample size in the study prevents drawing definitive conclusions.

It is difficult to ascertain whether a synergistic effect could be present given our study design and number of participants. Previous clinical experiences showed that the ketogenic diet may increase the effectiveness of radiation in pancreatic and lung cancer, but the long-term interactions between radiotherapy and metabolic targeting in GBM are unknown (41). As far as we know, there are no studies evaluating the synergy between the ketogenic diet and standalone chemotherapy in patients with GBM. Several experimental and clinical reports explored the combination of chemotherapy and ketogenic diets in other forms of cancer. For example, in a recent experimental study, Yang et al. (42) demonstrated that the ketogenic diet sensitizes murine pancreatic cancer tumors to cytotoxic chemotherapy. There is limited but encouraging clinical data regarding the co-administration of a ketogenic diet and chemotherapy in patients with neuroblastoma, as well as breast, pancreatic and gastric cancer (43–48).

Each of our patients differed in chemotherapy schedules. We wish to highlight patient 1, presenting with an IDH wild-type GBM, which has lived 84 months so far with an ECOG score 0. He has maintained the ketogenic diet as the only treatment modality for the last 5 years without recurrence and without chemotherapy. The remaining three surviving patients experienced recurrence and underwent chemotherapy again. Interestingly, no metabolic dysregulation was observed during chemotherapy and corticosteroid administration. More specifically, glucose levels did not exceed 80 mg/dL and satisfactory ketosis was maintained. It has been well established that patients undergoing corticosteroid administration show increased levels of glycemia, lactate and insulin, with an unfavorable effect on tumor progression (49–55). The administration of ketogenic diets during chemotherapy and/or corticosteroids may benefit cancer patients through the control of metabolic dysregulation, beyond direct effects on cancer cells.

Another noteworthy observation is that all patients with the longest survival were IDH mutant negative. IDH mutations have been associated with better prognosis in high-grade gliomas (IDH mutant grade 4 astrocytomas are no longer considered “true” GBM in the 2021 WHO classification). However, while long-term survivors have been reported in IDH “wild-type” GBM, the proportion of patients surviving over 5 years in unselected GBM populations is exceedingly small (56). It would therefore be expected that long-term survivors in our cohort would present IDH mutations, but this was not the case. Further studies will be needed to determine whether dietary KMT can benefit GBM patients regardless of IDH mutation status.

None of the patients who followed the diet developed dyslipidemia or reported major side effects. We suggest that the Mediterranean-type ketogenic diet may be suitable for at-risk patient populations such as cancer patients, offering more dietary flexibility than the classical 4:1 ketogenic diet used in epilepsy research (57).

Tumor recurrence despite diet adherence can be explained by secondary metabolic dependencies of tumor cells (58–61). It is important to acknowledge that dietary strategies alone, such as ketogenic diets or fasting, cannot fully deplete glucose from the tumor microenvironment. Dietary KMT intends to reduce pro-growth signaling, normalize the tumor microenvironment, and increase substrate competition between cancer and non-tumoral cells, but pharmacological metabolic targeting was not employed in this study. Glutaminolysis is recognized as the second major energy pathway in cancer and cannot be targeted using dietary strategies alone. Given that glutaminolysis was not targeted in this study, we hypothesize that glutamine dependence may have contributed to incomplete responses. Interestingly, some researchers disagree about the action of fats as cancer cell growth inhibitors and believe the opposite. Article by Sperry et al. concludes that glioblastoma utilizes fatty acids and ketone bodies for growth allowing progression during KMT therapy (62). Similar conclusions are drawn by an article by Duman et al. (63) demonstrating that the availability of fatty acyl-CoA into mitochondria is driving b-oxidation and promotes tumorigenesis. But both of the above mentioned papers are having a different goal from KMT. Their goal is to maintain SLP, and target FAO. The goal with KMT is to target SLP, and maintain FAO/OXPHOS. Consequently, proliferation rates will go down if fatty acid “utilization” is targeted. Fatty acids are used for biomass, when available (as per labeling), and partially “respired.”

Furthermore (64), radiation therapy may contribute to secondary metabolic reprogramming, increased tissue inflammation and heightened aggressiveness of the recurrent GBM cells, despite short-term cytotoxic effects. Given the design of this study, with all patients undergoing conventional cycles of radiotherapy, it is not possible to discern whether surviving cancer cell populations may have displayed different metabolic responses if chemotherapy was administered as a standalone therapy.

The encouraging results of our adhering participants highlight the importance of patient retention in ketogenic diet studies, with focus on palatability, reducing diet restrictiveness by leveraging modern food technologies (e.g., alternative recipes to common high-carbohydrate foods; non-caloric, non-insulinogenic sweeteners), and avoiding participant drop-out by dedicated nutritional support.

Patients with GBM experience reduced quality of life due to the disease itself, surgical resection and intensive chemoradiotherapeutics regimens. As a consequence, it may be difficult to introduce overwhelming changes to their daily routines, food choices or lifestyle habits (65–67). It is therefore essential to describe the scientific rationale behind dietary KMT to both the patient and their caregivers to improve diet adherence, fostering patient autonomy.

Another challenge for diet implementation is finding compatibility with conventional treatments and traditional dietary advice for cancer patients. To make the application of ketogenic diets more widely accessible, it is fundamental for both the patient and their treating oncologist to discuss the available experimental and clinical data on a case-by-case basis. A review published by Valerio et al. (68) included all prospective, retrospective, and randomized clinical studies, as well as case reports on the use of KD as an adjunctive therapy for primary brain tumors, including recurrent and newly diagnosed GBM. These studies exhibited significant heterogeneity in their design, tumor type, diet implementation and duration, number of participants, patient demographics, and outcome measures. Consequently, comparing the results of these studies with one another—and with our own study, which focused exclusively on patients with newly diagnosed GBM—presents substantial challenges. Overall, KD was generally well-tolerated across the studies, although adherence proved difficult due to the diet’s restrictive nature. Survival outcomes varied, with some studies indicating potential benefits in specific subgroups, such as younger patients or those with favorable genetic markers like IDH1 mutations. Median overall survival (OS) across the studies ranged from 8 to 60 months, with the majority reporting improved overall survival compared to the known median survival time associated with standard therapy for GBM. However, small sample sizes and methodological differences limit the broader applicability of these results. Undoubtedly, further research involving larger, more homogeneous patient cohorts and standardized ketogenic diet protocols is needed to reach more definitive conclusions. To summarize, an in-depth evaluation of the scientific literature, which shows beneficial effects on survival, quality of life and self- efficacy, is essential prior to starting this intervention (10, 13, 16, 24, 69–73).

Lastly, interventional dietary studies require special attention to methodology and planning to ensure the patient is well-informed and eventually able to make decisions regarding food choices without constant guidance. This is self-evident if we consider that the “diet intervention” is expected to last several years. Accordingly, it will be important to integrate dietary KMT as part of the standard of care, rather than relying on patient autonomy to externalize it as a complementary treatment modality. It is important to mention that the suitability of the ketogenic diet should be evaluated by a dietitian, neurologist, oncologist and/or neurosurgeon specializing in ketogenic diets. The number of healthcare professionals familiar with nutritional ketosis and its practical applications is growing due to renewed interest in metabolic interventions for chronic diseases (74). This will facilitate adherence in future clinical studies, building from individual clinical experiences and moving into larger clinical trials.

## 6 Conclusion

In our study, patients who followed a well-designed ketogenic diet had longer survival compared to patients who did not follow it. Although the number of patients is small, the results are encouraging and motivate us to plan studies with a larger number of patients, where we will examine the effects of the diet separately in women and men. Furthermore, when the ketogenic diet was applied alongside chemotherapy and corticosteroids, no dysfunction in glucose metabolism was observed. This is very important and perhaps the deepest message of our work, as we know that cancer cells exhibit altered and increased glucose uptake and increased glycolysis, which was first recognized by Otto Warburg 70 years ago (7, 8). It is also well known that both chemotherapy and steroids increase glycolysis (75, 76). Therefore, on one hand, they have a beneficial effect, but on the other hand, they promote the growth of cancer cells by increasing glycolysis (64, 77). And this is precisely where the important contribution of the ketogenic diet lies, which, as shown by our results, helps to prevent the metabolic dysregulation resulting from conventional treatment for glioblastoma (78–81). The ketogenic diet, however, could have other contributions to the treatment of these patients. Brain tumors rely energetically on glycolysis, unlike healthy neurons/glial cells, which can alternatively consume ketone bodies. Thus, ketone bodies have a toxic effect on cancer cells while leaving healthy cells unaffected. So, it seems that the mechanisms of diet work at multiple levels, thus helping to prolong the survival of these patients. The number of patients in our study is small, but it gives us an important incentive to perform larger studies with a larger number of patients, in the hope of providing another weapon in the treatment of this incurable brain tumor.

## Data Availability

The raw data supporting the conclusions of this article will be made available by the authors, without undue reservation.
