# Corrigendum: Successful application of dietary ketogenic metabolic therapy in patients with glioblastoma: a clinical study

**DOI:** 10.3389/fnut.2025.1614194

**Published:** 2025-05-19

**Authors:** Andreas Kiryttopoulos, Athanasios E. Evangeliou, Irene Katsanika, Ioannis Boukovinas, Nikolaos Foroglou, Basilios Zountsas, Angeliki Cheva, Vaios Nikolopoulos, Thomas Zaramboukas, Tomas Duraj, Thomas N. Seyfried, Martha Spilioti

**Affiliations:** ^1^Department of Neurology, Aristotle University of Thessaloniki, Thessaloniki, Greece; ^2^Division of Child Neurology, St Luke's Hospital, Thessaloniki, Greece; ^3^Department of Diet and Nutrition, Papageorgiou General Hospital, Thessaloniki, Greece; ^4^Bioclinic Thessaloniki Medical Oncology Unit, Thessaloniki, Greece; ^5^Department of Neurosurgery, Aristotle University of Thessaloniki, Thessaloniki, Greece; ^6^Department of Neurosurgery, St Luke's Hospital, Thessaloniki, Greece; ^7^Department of Pathology, Faculty of Medicine, Aristotle University of Thessaloniki, Thessaloniki, Greece; ^8^ISTODIEREVNITIKI S.A., Surgical Pathology and Cytopathology Laboratories, Thessaloniki, Greece; ^9^Department of Biology, Boston College, Chestnut Hill, MA, United States

**Keywords:** ketogenic, glioblastoma, diet, multiforme, metabolic, brain, tumor

In the published article, there was an error in Figure 10 as published. An earlier version of Figure 10, containing outdated data was inadvertently resubmitted during the revision process. We sincerely apologize for this oversight and respectfully request that a corrigendum be issued to replace the incorrect figure with the correct one.

The corrected Figure 10 and its caption appear below.

**Figure 10:**
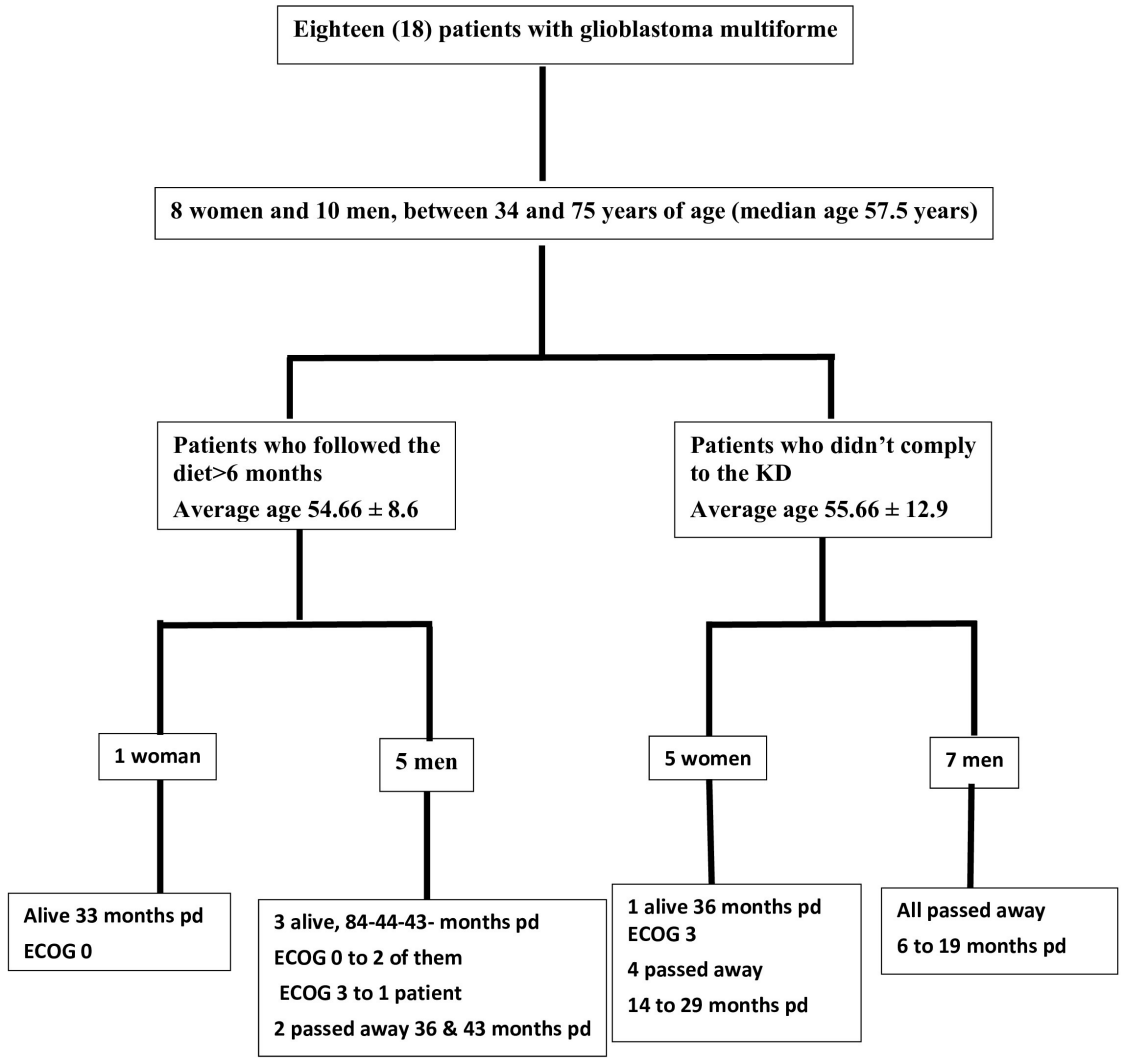
Study flow chart. pd, post diagnosis; ECOG, Eastern Cooperative Oncology Group.

The authors apologize for this error and state that this does not change the scientific conclusions of the article in any way. The original article has been updated.

